# A new label free spiral sensor using impedance spectroscopy to characterize hepatocellular carcinoma in tissue and serum samples

**DOI:** 10.1038/s41598-024-63141-5

**Published:** 2024-06-07

**Authors:** Reda Abdelbaset, Sherif M. Shawky, Mohammed A. A. Abdullah, Omar E. Morsy, Yahia A. Yahia, Yehya H. Ghallab, Marwa Matboli, Yehea Ismail

**Affiliations:** 1 Biomedical Engineering Department, Helwan University, Cairo, 11795 Egypt; 2 Centre of Nanoelectronics and Devices (CND), The American University in Cairo (AUC), New Cairo, 11835 Egypt; 3 Centre of Nanoelectronics and Devices (CND), Zewail City of Science and Technology, Giza, 12588 Egypt; 4 Biochemistry Department, Faculty of Pharmacy, Misr University for Science and Technology, Giza, 12566 Egypt; 5 Center of Genomics, Helmy Institute, Zewail City of Science and Technology, Giza, 12588 Egypt; 6 Biochemistry and Molecular Biology Department, Faculty of Medicine, Ain Shams University, Cairo, 11591 Egypt

**Keywords:** Electrical impedance spectroscopy (EIS), Phase angle, Capacitive sensor, Hepatocellular carcinoma, Printed circuit board (PCB), Serum, Tissues, Biochemistry, Cancer, Biomarkers, Health care, Medical research, Engineering

## Abstract

Hepatocellular carcinoma (HCC) stands as the most prevalent form of primary liver cancer, predominantly affecting patients with chronic liver diseases such as hepatitis B or C-induced cirrhosis. Diagnosis typically involves blood tests (assessing liver functions and HCC biomarkers), imaging procedures such as Computed Tomography (CT) and Magnetic Resonance Imaging (MRI), and liver biopsies requiring the removal of liver tissue for laboratory analysis. However, these diagnostic methods either entail lengthy lab processes, require expensive imaging equipment, or involve invasive techniques like liver biopsies. Hence, there exists a crucial need for rapid, cost-effective, and noninvasive techniques to characterize HCC, whether in serum or tissue samples. In this study, we developed a spiral sensor implemented on a printed circuit board (PCB) technology that utilizes impedance spectroscopy and applied it to 24 tissues and sera samples as proof of concept. This newly devised circuit has successfully characterized HCC and normal tissue and serum samples. Utilizing the distinct dielectric properties between HCC cells and serum samples versus the normal samples across a specific frequency range, the differentiation between normal and HCC samples is achieved. Moreover, the sensor effectively characterizes two HCC grades and distinguishes cirrhotic/non-cirrhotic samples from tissue specimens. In addition, the sensor distinguishes cirrhotic/non-cirrhotic samples from serum specimens. This pioneering study introduces Electrical Impedance Spectroscopy (EIS) spiral sensor for diagnosing HCC and liver cirrhosis in clinical serum—an innovative, low-cost, rapid (< 2 min), and precise PCB-based technology without elaborate sample preparation, offering a novel non-labeled screening approach for disease staging and liver conditions.

## Introduction

Hepatocellular carcinoma (HCC) is a serious global health issue, accounting for the fourth leading cause of death in the world, with around 905,677 new cases and 830,180 deaths in 2020^1,2^. According to the number of liver cancer patients, Egypt is second in the world in 2020^3,4^. Currently, liver biopsy is the gold standard for detecting HCC^5^, and it is utilized to distinguish between normal and malignant tissues. However, it has limitations such as invasiveness, high cost, time-consuming, and labor-intensive procedures, all of which can lead to major consequences during and/or after the surgery^6,7^. Controlling and managing HCC is largely dependent on accurate diagnosis and prognosis, which is difficult to achieve because HCC is typically asymptomatic until late in the illness, with several clinical consequences. Therefore, there is an urgent need for the development of novel methods for prognosis, and treatment follow-up for HCC, especially in the high-risk population.

Biological tissues exhibit electrical Impedance that varies with frequency. Various tissues and hence, cells contain distinctive components with both resistive and capacitive properties resulting in a complex electrical impedance. The magnitude of impedance along with the dependence of its frequency is a function of the cell composition. Consequently, by measuring the impedance of cells across a wide range of frequencies, a spectrum will be generated; that is specific to the measured cells and/or tissues. Therefore, the change in the impedance spectrum is directly related to the change in the cells or tissue nature including its components, population, dimensions, and arrangements^8^. The impedance measured from biological samples to the passage of an electric field in the frequency spectrum, which can represent the physiological state of cells, is referred to as electrical impedance spectroscopy (EIS)^9^. The capacitance of the cell membrane and the resistance of the cytoplasm make up the equivalent impedance of a single cell. The electrical characteristics of the cell are influenced by the composition of the cell membrane, cytoplasm components/cargo, and the intracellular and intercellular space. Thereby, discrimination between different types of cells including normal and malignant^10^. Activation of biological cells and/or tissues at different frequencies, they exhibit different electrical properties, regarding resistance, conduction, and permittivity thus, EIS in medicine and biology has many advantages such as label-free, non-invasiveness, cost-effective, portability, and ease of use^10,11^. For these reasons, cell electrical characteristics have been studied and applied in a wide range of domains, including disease diagnosis^12,13^ environmental monitoring^14^, food safety^15^, and cell identification and separation^16^. Electrical impedance spectroscopy has been utilized to investigate cell electrical properties^17,18^.

Impedance spectroscopy is a quick, non-destructive, and easily automated method for studying the electric characteristics of a wide range of materials after applying a sinusoidal signal. Impedance spectroscopy is a widely used technique for the characterization of biological cells. Herein, an impedance spectroscopy platform for detecting HCC in serum and tissues has been developed using a novel spiral electrode, which is implemented using printed circuit board (PCB) technology. The proposed platform has been successfully used in proper discrimination between the HCC and normal subjects in clinical tissue samples exploiting the different electrical properties response to electrical impedance spectroscopy (EIS) at different frequencies between normal and HCC cells. Our hypothesis in EIS discrimination in serum samples is based on the differential expression of nucleic acids, proteins, and extracellular vesicles (exosomes, HCC signature), resulting in the specific and sensitive detection of HCC compared to the control group at specified frequencies in less than 2 min. The HCC grade, cirrhotic/non-cirrhotic samples have been differentiated from serum specimens. To the best of our knowledge, this is the first study using EIS based for the diagnosis of HCC, and liver cirrhosis in clinical serum specimens.

The main objective of this study is to establish a novel impedance spectroscopy platform, based on PCB technology, for the direct detection of HCC in real clinical tissues, and serum specimens, compared to healthy subject controls. In this stage of the study, we used 24 tissues and sera clinical samples as proof of concept. Moreover, differentiation between HCC stages (according to The Barcelona Clinic Liver Cancer staging system (BCLC)), cirrhotic/non-cirrhotic liver in clinical tissues and serum samples respectively. To the best of our knowledge, this is considered the first PCB based on EIS technology used in the direct non-labeled diagnosis of HCC in serum specimens.

## Methodology

**Figure 1:**
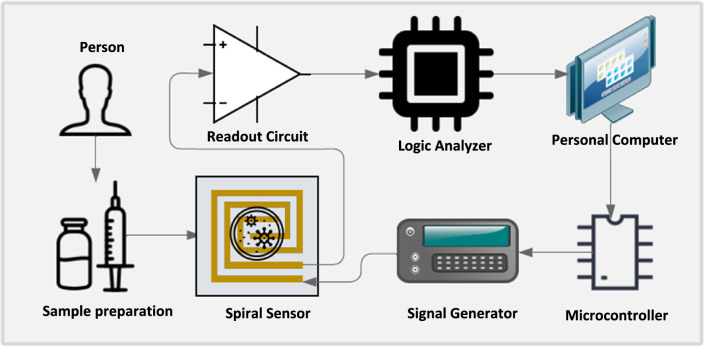
The flow work of the proposed platform.

The concept of the proposed biochip is based on measuring the impedance of the sample throughout the sensor, which is made up of two spiral electrodes wrapped around one another, one to generate an electric field and the other to sense the field penetrating through the sample. This value is then converted into a voltage by the readout circuit, which is converted into a digital format by the data acquisition so that it is processed by the personal computer and used to compute the electrical properties based on mathematical equations for precise differentiation between the tested samples. The next sections, go into further depth on each component of the proposed platform. Figure 1 depicts a simplified schematic of the proposed platform that consists of a spiral EIS microelectrode, function generator, readout circuit, data acquisition, and personal computer.

The tested samples (20 μL) are directly applied to the sensor using a micropipette, and then a 3 voltage peak-to-peak (Vpp) of a certain range (100 kHz to 3 MHz) of frequencies of AC sine wave signal are applied to one of the dual spiral microelectrodes to calculate the dielectric properties (i.e. impedance, capacitance, permittivity, and conductivity) of the tested sample using a MATLAB algorithm that solves a set of equations (explained below) based on the measured amplitude of the other electrode of the dual spiral microelectrode through the readout circuit and the phase shift between the input (applied)/the output (measured) signals. However, these electrical aspects are preferred to achieve the best performance of the proposed sensor without using a passivation layer which affect the measuring of the dielectric properties of the tested samples as mentioned in previous work^17,19^.

### Microelectrode

**Figure 2:**
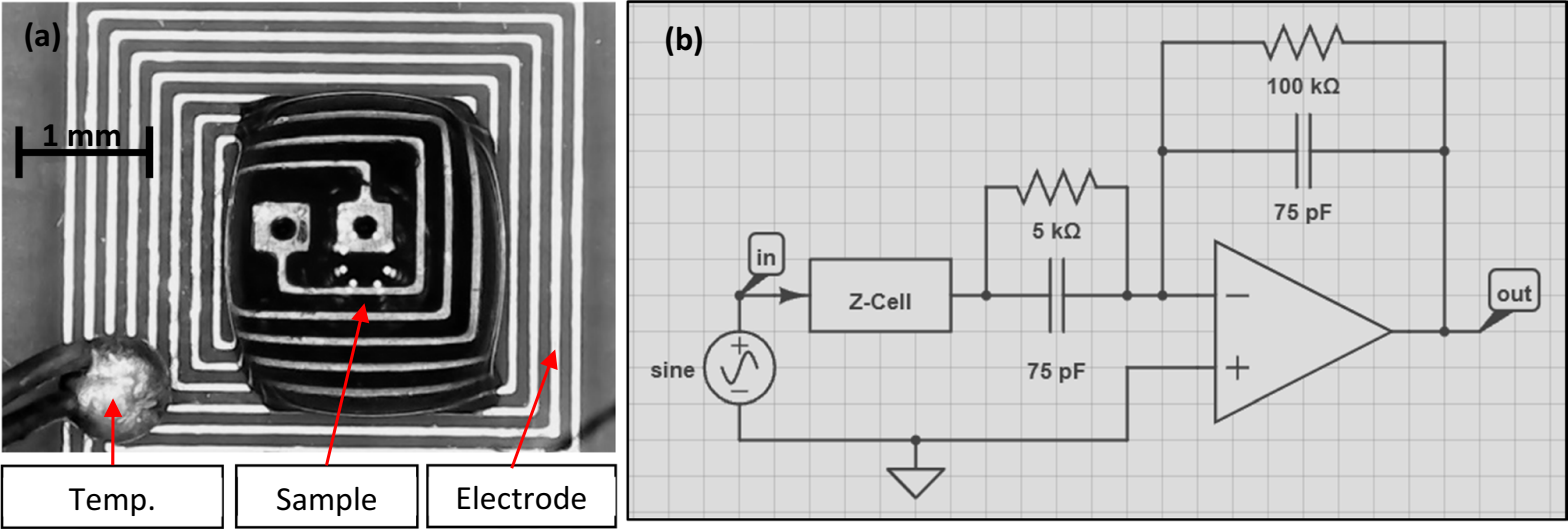
(**a**) The fabricated EIS microelectrode, and (**b**) The readout circuit of the impedance spectroscopy.

A unique design for a pair of spiral electrodes has been done for measuring impedance as illustrated in Fig. 2a, and the snapshot of the CAD file showed in (Fig. 1 Supplementary Information). The spiral shape provides additional benefits over typical designs (i.e., longitudinal^20,21^, bi-cross^21^, tri-cross^22^, or multi-cross^23^), such as lengthy electrodes in a compact area, which facilitates the penetration of the electric field to the tested sample to the sensing electrode from all directions. PCB technology is used to implement the proposed design with the following specifications: A double layer using substrate (0.062ʹʹ ± 10%, FR4-TG130). The electrode thickness is 1 oz. (34.79 μm). The proposed microelectrode design consists of a dual spiral, each spiral electrode has five turns with a width of 100 μm and a spacing of 100 μm. However, 100 μm is preferred for spacing and width regarding the minimum dimension can be achieved due to the fabrication limitations. These dimensions are chosen to accommodate the fabrication constraints and standards. The suggested microelectrode is made of copper and immersed in silver for biocompatibility^24^. The fabricated PCB chip is printed by Gold Phoenix PCB, Oakville, Canada.

The appropriate signal sequences are given to the recommended EIS microelectrode utilizing our previously constructed signal generator architecture^25^. The needed signal parameters for this investigation are in a frequency range of 100 kHz–3 MHz and amplitude of 3 (Vpp) of AC-sine waves. A temperature sensor is used to measure the temperature increase at the spiral sensor that can occur because of applying the required voltage. Because of the low voltage used, the recorded temperature increase is quite minimal (0.1 ℃), with almost no effect on the detection.

### Readout circuit

A charge amplifier with a feedback capacitor Cf = 75 pF and a feedback resistor Rf = 100 k is used to convert the impedance change to a voltage output signal, as illustrated in Fig. 2b. To maintain a logarithmic output of the readout circuit, a parallel capacitor (Cf = 75) pF and a resistor (Rf = 5 k) is added to the input of the op-amp (AD9851). The signal from the readout circuit is acquired using a logic analyzer (Saleae Logic 8) with appropriate features up to 5 MHz frequency^20,21^.

### Data processing

The personal computer is used extensively in this work to operate, monitor, analyze, and evaluate the proposed EIS biochip. Using LabVIEW, a user interface is created to operate the constructed function generator and receive the output alternating current (AC) signal from the function generator as well as the output AC signal from the readout circuit via the data acquisition^22^. The user interface also calculates the phase difference between the input and output signals. Finally, export the amplitudes of the input and output signals of the EIS microelectrode, as well as the phase shift, to an Excel file.

A MATLAB algorithm is used to transform, monitor, and assess the experimental readings from the EIS biochip. The following are the governing equations for calculating dielectric properties depending on the amplitude of input/output signals and the phase shift between them:

1 $${x}_{1 }= \frac{-{A}_{i}{R}_{f}}{{A}_{o}\sqrt{1+{\left(2\pi f{C}_{f}{R}_{f}\right)}^{2}}},$$

2 $${x}_{2 }= \frac{-{A}_{i}{R}_{f}}{{A}_{o}\sqrt{1+{\left(2\pi f{C}_{f}{R}_{f}\right)}^{2}}},$$

3 $${Q}_{1}= -ph- {\text{tan}}^{-1}\left(2\pi f{C}_{f}{R}_{f}\right),$$

4 $${Q}_{2}= - {\text{tan}}^{-1}\left(2\pi f{C}_{f}{R}_{f}\right),$$

5 $${R}_{z}= {x}_{1}\text{ cos}\left({Q}_{1}\right)+ {x}_{2}\text{ cos}({Q}_{2}),$$

6 $${I}_{z}= {x}_{1}\text{ sin}\left({Q}_{1}\right)+ {x}_{2}\text{ sin}({Q}_{2}),$$

7 $$Impedance\, magnitude \left|Z\right|\left(\Omega \right)= \sqrt{{R}_{z}^{2}+{I}_{z}^{2}},$$

8 $$Impedance \,angle= {\text{tan}}^{-1}\frac{{I}_{z}}{{R}_{z}},$$

9 $$Capacitance\, \left(Farad\right)= \frac{1}{2\times \pi \times F\times {I}_{z}},$$

where Ai is the amplitude of the applied signal, Rf is the resistance of the feedback resistor of the readout circuit, A0 is the amplitude of the output signal of the readout circuit, f is the frequency of the applied signal, Cf is the capacitance of the feedback capacitor of the readout circuit, ph is the phase shift between the applied and output signal.

Additional factors are estimated to improve the differentiate between normal and abnormal samples such as the dielectric properties (permittivity and conductivity). The permittivity is calculated in the following form^22^:

10 $$\varepsilon = \frac{C d}{{\varepsilon }_{0}A},$$

where C is the capacitance of the tested cell, d is the thickness of the cell membrane, A is the area of the tested cell, and ${\varepsilon }_{0}$ is the permittivity of the space. While the conductivity is estimated as follows^23,25^.

11 $${\text{t}}={\text{RC}},$$

12 $$\sigma = \frac{t}{A} . \frac{real(Z)}{{real(Z)}^{2}+ {imaginary (Z)}^{2}},$$

where t is the relaxation time, R is the resistance of the tested cell, real (Z) is the real part of the impedance of the tested cell, and imaginary (Z) is the imaginary part of the impedance of the tested cell.

### Biological samples

The study involved procuring tissue samples from both control subjects and patients diagnosed with hepatocellular carcinoma (HCC), following the practical guidelines established by the American Association for the Study of Liver Diseases (AASLD).

Written informed consents were obtained from all participants according to the Declaration of Helsinki. This work was approved by the Faculty of Medicine Ain Shams University Research Ethical Committee, Egypt (FMASU R 38/2021). The study involved 24 paired fresh liver tissue specimens & sera samples from each participant (as a proof of concept) following the practical guidelines established by the American Association for the Study of Liver Diseases (AASLD) that were collected from internal medicine and general surgery department in Ain Shams University hospitals during the period between April 2021 and May 2022. Explanted HCC tissue samples obtained from the tumor of patients who underwent curative resection of all tumor nodules and the cut surface being free of cancer by histologic examination. For comparison, Normal liver tissue samples were also obtained from corresponding non-tumor fresh specimens from the same individuals. The diagnosis of HCC was histologically confirmed, and only HCC tumors with a percentage of tumor cells more than 90% and without extensive necrosis were used for the analysis. The degree of cirrhosis was also assessed by the pathologist. Also, the control liver tissue was verified to have normal liver architecture. Seven patients with chronic HCV sera samples and six-healthy controls sera samples were recruited during their routine medical checkups with normal liver function and negative viral markers. Diagnosis of HCV was proved by positive anti-HCV antibodies and RNA of HCV by PCR.

Tissue Bio-specimen collection was performed by both the surgeon and pathologist. Fresh liver tissues were cleaned twice with sterile PBS and were cut into smaller slices in a metal mold and then aliquoted in labeled cryovials. The samples were transferred to pathology and biochemistry labs to be processed within 2 h. Liver tissues in these vials were kept at 4 °C on ice at 4 °C for 2 h and later transferred to minus 70 °C freezing for long-term storage. Tissue processing was executed aseptically in a Class II biosafety cabinet. Quick disaggregation of the deep-frozen tissue (to avoid thawing) before analysis by mincing into small pieces (~ 1 mm) and then washing in sterile PBS medium to remove loosely bound cells or non-specific debris by gentle agitation & repeated aspiration through pipettes^26^. Blood samples were collected and centrifuged at 4000 rpm for 20 min to obtain the serum and were liquated (each sera samples were divided into 2 smaller aliquots to decrease the freeze–thaw cycle effect). All sera samples were stored at − 80 ℃ for further processing.

Various biological tests were conducted to assess liver function and hepatocyte integrity, including measurements of bilirubin, albumin, international normalized ratio (INR), aspartate aminotransferase (AST), and alanine aminotransferase (ALT). Importantly, none of the patients had undergone radiation, chemotherapy, or surgery before enrollment in the study. The study was conducted on 24 HCC patients, and 13 control individuals (Supplementary Information Table 1). The mean age of the two groups was 57.5 ± 7.6 for the HCC group and 53.85 ± 4.9 for normal patients. In the HCC group, non-cirrhotic patients were four non-cirrhotic patients, while there were 5 cirrhotic patients in the control group. In the hepatocellular carcinoma (HCC) group (N = 24), there was a statistically significant increase in the levels of aspartate aminotransferase (AST) (mean ± SD: 69.2 ± 35.65296 U/L) and alanine aminotransferase (ALT) (mean ± SD: 49.2 ± 24.05696 U/L), compared to the control group (N = 13) with means of 46.61 ± 24.801 U/L for AST and 31.76 ± 16.83327 U/L for ALT, respectively (P-value = 0.031 for AST, P-value = 0.014 for ALT). Additionally, albumin levels were significantly lower in the HCC group (mean ± SD: 2.96 ± 0.354985 g/dL) compared to the control group (mean ± SD: 3.4 ± 0.543139 g/dL) (P-value = 0.017). Furthermore, the international normalized ratio (INR) was significantly higher in the HCC group (mean ± SD: 1.358 ± 0.190086) compared to the control group (mean ± SD: 1.169 ± 0.127375) (P-value = 0.001). Notably, the concentration of alpha-fetoprotein (AFP) exhibited a substantial escalation in the HCC group (mean ± SD: 335.4 ± 768.2474 ng/mL) compared to the control group (mean ± SD: 6.46 ± 5.825453 ng/mL), yielding a statistically significant difference (P-value = 0.047).

The HCC severity is generally classified The Barcelona Clinic Liver Cancer (BCLC) Staging System is a widely employed classification for primary liver cancer, providing a comprehensive framework for staging and treatment planning. It assesses the extent of cancer spread within the liver or to other parts of the body, the functioning of the liver, the overall health and wellness of the patient, and the symptoms caused by the cancer. The BCLC system categorizes liver cancer into five stages, ranging from very early (Stage 0), stage A, stage B, stage C to end-stage (Stage D), offering a valuable tool to predict patient recovery chances and guide appropriate treatment strategies^27^. According to the BCLC system, the A stage is the early stage while the D stage is the most severe.

### Handling sample and its application to the sensors

Tissue samples were placed in Petri plates and prepared using a scalpel before being suspended in a saline medium. A 20 μL sample was then introduced onto the electrode for analysis. Similarly, serum samples involved the application of a 20 μL volume onto the electrode surface for analysis. Each sample was tested in three trials and the standard error was estimated at each point for three trails of 24 samples using the following formula^28^:

13 $$SE= \frac{SD}{\sqrt{n}},$$

where SE is the standard error, SD is the standard deviation of measured values at a certain frequency, and n is the number of tested samples.

The dielectric properties of both normal and HCC (Hepatocellular Carcinoma) tissues and serum were measured using the spiral electrode. The spiral electrode was employed to detect HCC by analyzing impedance and phase angle measurements in both cellular and serum samples from HCC and control groups.

### Ethics approval and consent to participate

All participants provided written informed permission by the Declaration of Helsinki. The Research Ethical Committee of the University of Ain Shams Medical School in Egypt approved this work.

## Results

### Sensor validation

**Figure 3:**
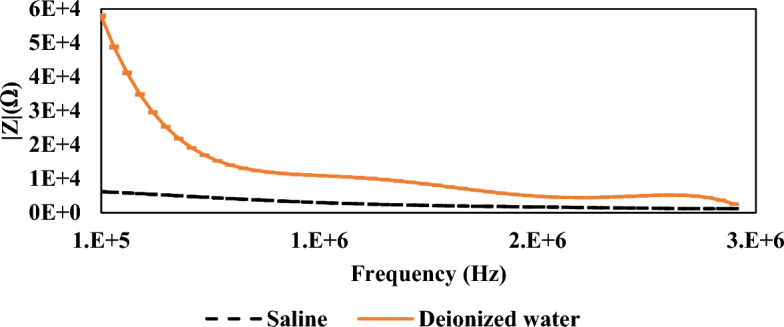
The magnitude and angle of the measured impedance of two media saline and deionized water.

In order to validate the sensor’s accuracy, a test was conducted using two distinct standard samples: deionized water and saline solution (the sample media). The purpose was to analyze the variation in impedance between these two standard samples. Figure 3 illustrates the disparity in impedance magnitude between saline (depicted by the dashed line) and deionized water (represented by the solid line) across a frequency range from 100 kHz to 3 MHz. The curve clearly indicates that the impedance of deionized water is higher than that of saline, which aligns with expectations. This discrepancy is anticipated due to the lower conductivity of deionized water compared to saline, which contains ions facilitating electrical current.

The impedance is varied according to frequency. At low frequencies, the magnitude of impedance of saline and deionized water varies significantly. A complex number can be used to represent impedance. The real part denotes a resistive component in the load that may be detected at low frequencies. The imaginary part denotes a capacitive or inductive component in the load. As saline contains more ions than deionized water, the resistance of saline was lower than that of deionized water, which demonstrates the efficiency of the spiral sensor. In general, the EIS in biological samples can be expressed by the flow of electrical field at different frequency range, and the resistance and reactance of this field flow by the biological specimen under investigation, which by measuring the impedance of the physiological state of the sample could be determined.

The phase angle is one of the well-established Bioelectrical impedances in clinical research and has a significant role in the evaluation and prognosis of different clinical disorders including cancer^29^. It is a mirror and contributes to the cell’s resistance (cytoplasm) and reactance (cell membrane) in which it is positively associated with reactance and negatively associated with resistance^30^. A low phase angle indicates cell death or decreased cell integrity, while high values are a sign of large quantities of intact cell membranes, as well as hydration and well nutritional status^31^. Many research groups have used Phase angle as prognosis and mortality rate in different cancers such as breast cancer^32^, colorectal cancer^33^, and non-small cell lung cancer^34^. Also, in a recent meta-analysis study, and the authors have concluded that the phase angle is considered as an important prognostic factor in survival of cancer patients^35^ as well as other clinical disorders such as liver cirrhosis^36^.

As mentioned above, the phase angle is directly proportional to the large quantities of intact cells and vice versa. Consequently, the obtained data could be explained by the fact of huge difference in size between cells which ranges from 10 to 50 microns^37^, in normal cells compared to cancer cells Interestingly, the phase angle of the cancer cells is much lower than the normal cells, which confirms the difference between the two cells, such as lower cell integrity, and large nucleus/cytoplasm ratio, and high capacitance of HCC cells. In the same context, the normal cells have a high phase angle as they are opposed to the cancer cells as mentioned before.

### Characterizing of normal liver tissues from hepatic cellular carcinoma

**Figure 4:**
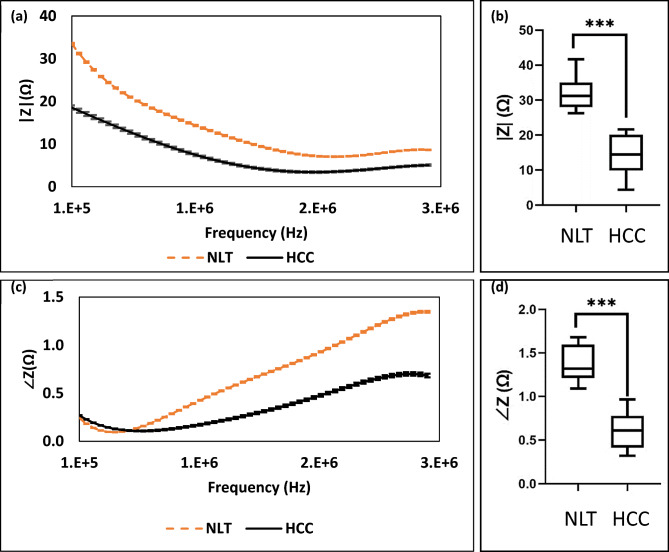
(**a**) The impedance amplitude of normal liver cells (NLC) and HCCs versus frequency. (**c**) The phase angle of normal liver cells and HCCs versus frequency. (**b**) The t-test and nonparametric test (Mann–Whitney Test) of impedance amplitude of NLT and HCT at 100 kHz (P-value < 0.0001), and (**d**) the phase angle of NLC and HCC at 3 MHz (P-value < 0.0001), where * equal P-value < 0.05.

The magnitude of impedance and phase angle was assessed for both normal liver tissues (NLT) and hepatocellular carcinoma (HCC) across a frequency range from 100 kHz to 3 MHz, as depicted in Fig. 4. Notably, at lower frequencies, especially at 100 kHz (as seen in Fig. 4a), there is a substantial difference in the impedance magnitude between normal liver tissues and HCCs. As the frequency increases, the discrepancy in impedance magnitude diminishes, yet remains notably significant. Contrary to the impedance magnitude, the impedance angle showcases a distinct behavior. Initially, the phase angle difference between NLT and HCC is almost negligible, but it escalates with increasing frequency, reaching its pinnacle at 3 MHz, as depicted in Fig. 4c.

Using a t-test and nonparametric test (Mann–Whitney Test), the differences in the impedance amplitudes and phases of all tissues and serums of normal and HCC subjects were evaluated using (Graph pad Prism 9 software) as shown in Fig. 4b,d. The impedance amplitudes of normal liver cells and HCCs were measured at 100 kHz, while the control serum and HCC serum samples were compared at 400 kHz. The statistical results show a three-star significant difference in impedance amplitude between normal liver cells and HCCs at 100 kHz, with a p-value of 0. 001. Additionally, the statistical results show a three-stars significant difference in the impedance phase angle of normal liver tissues (NLT) and HCC at 3 MHz with a P-value < 0.0001.

### Characterizing of normal serum samples from hepatocellular carcinoma serum samples

**Figure 5:**
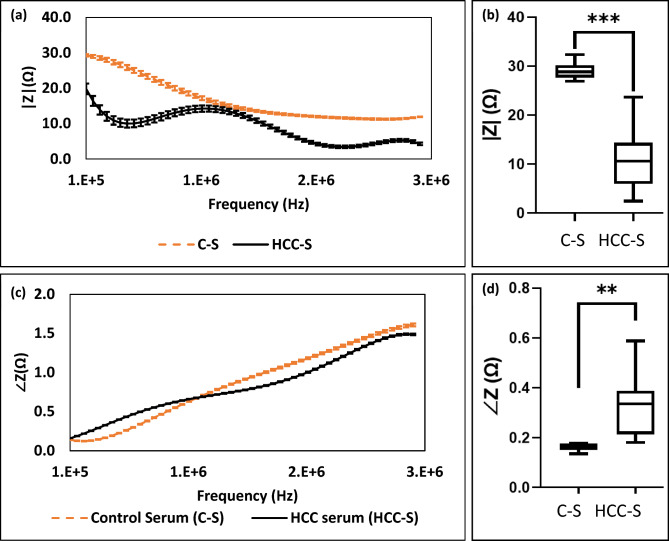
(**a**) The impedance amplitude of control serum and HCC serum versus frequency, (**c**) the phase angle of control and HCC serum versus frequency. (**b**) The t-test and nonparametric test (Mann–Whitney Test) of the impedance amplitude at 400 kHz (P-value: 0.0003), (**d**) the phase angle of control and HCC serum at 400 kHz (P-value: 0.0044).

The serum samples from healthy and HCC patients were tested using the same spiral sensor. As illustrated in Fig. 5, generally, in both healthy and HCC serum samples, the impedance magnitude decreases with an increase in the frequency range of 400 kHz and 3 MHz respectively. There is a noticeable distinction between the two: the impedance magnitude of healthy serum is notably higher than that of the HCC sample, indicating a significant disparity between these sample types (Fig. 5a). The impedance magnitude of healthy serum consistently decreases with increasing frequency. However, in the case of the HCC sample, a wavering pattern emerges. While the overall trend indicates a decrease with rising frequency, it does not follow a consistent decline. Nonetheless, despite this fluctuating behavior, there is a general tendency towards reduced impedance with higher frequencies in the HCC sample. In Fig. 5c, the phase angle trends of both the healthy and HCC samples show a general increase with rising frequency. However, these two trends overlap, causing the curves to lack distinctiveness for each group. Consequently, they cannot be effectively utilized to differentiate between the healthy and HCC samples. Therefore, the designed spiral sensor was able to discriminate between the HCC and normal groups in both types of samples (cells and serum); regarding the impedance amplitude and phase angle at 400 kHz.

### Cirrhosis, and BCLC stage measurements

**Figure 6:**
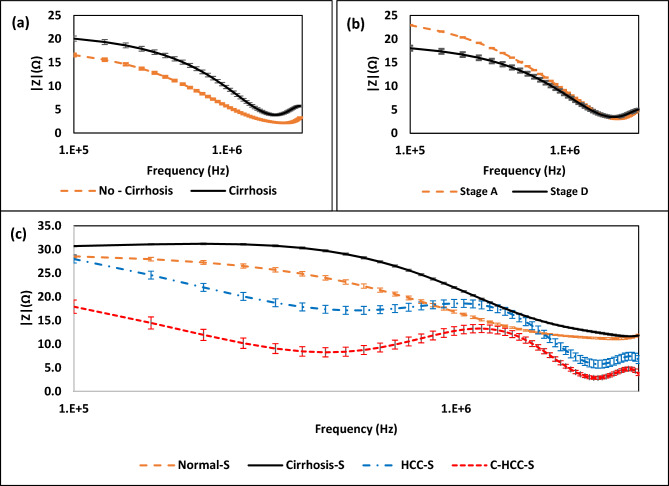
The impedance amplitude of: (**a**) No-cirrhosis and cirrhosis HCC tissues, (**b**) stage A, and stage D HCC tissues, and (**c**) normal, cirrhosis, HCC, cirrhosis HCC from serum.

The spiral sensor was used to discriminate between healthy tissues and cirrhotic tissues. As shown in Fig. 6a, the cells with cirrhosis have a greater impedance amplitude than the normal samples. This means that when fibrosis progresses, cell structure changes and resistance to the current flow occurs ending with increasing the impedance magnitude. Continuously, as shown in Fig. 6b, the samples with BCLC stage D have a lower impedance amplitude than the samples with stage A. This indicates that as the stage develops, cell activity increases, resulting in a lot of charges, shrinkage in the cancer cell, which will be discussed in detail later, and a small impedance magnitude observed in the cells. Furthermore, the spiral sensor was used to discriminate between healthy, cirrhosis, and cirrhosis HCC serums. As shown in Fig. 6c, the serums with cirrhosis have a greater impedance amplitude than the normal and HCC samples. These results confirm the results of tissues.

### Dielectric properties of the tested samples

**Figure 7:**
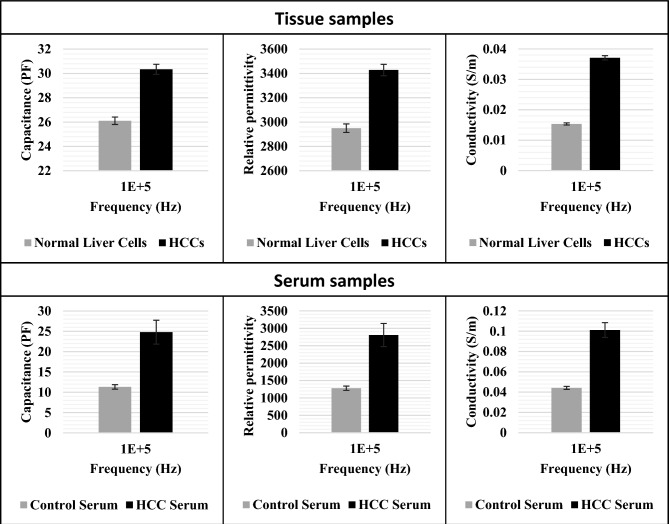
A bar graph of the dielectric properties of normal liver tissues (NLTs) and HCCs at 100 kHz (P-value: 0.0032), and control serum (C-S) and HCC serum (HCC-S) at 400 kHz: (**a**) capacitance, (**b**) permittivity, and conductivity. Where, ε0  ≈  8.85 × 10^−12^  F/m^38^, d = 10^−4^ m^39^, and A = 4.84 × 10^−9^ m^2^.

The dielectric properties of the HCC and normal cells/serum have been measured. As shown in Fig. 7, the capacitance, permittivity, and conductivity of tissues and serum are estimated at 100 kHz which has proven to be the best among all frequencies. There is a significant difference in the capacitance, permittivity, and conductivity of tissues and serum in both groups. Unlike the impedance of HCC, which is lower than that of control tissues and serum, the capacitance, permittivity, and conductivity are markedly higher than those of the control tissues and serum.

Our results align with the findings reported by Laufer et al., who utilized the four-electrode method to measure the electrical impedance of hepatic tumors and adjacent normal liver tissues on ex vivo intact liver^40^. As shown in Table 1, a comparison between the Four-electrode method and our spiral method reveals at a frequency of 100 kHz that the impedance in HCC is smaller than normal in both methods, while the conductivity of HCC is higher than normal. Additionally, the permittivity in HCC for both methods is higher than in normal tissue. Notably, both results of dielectric measurements align with a lower error bar in our spiral method, indicating greater precision. Furthermore, our method allows application on tissues and serum samples, providing practical convenience compared to the ex vivo intact liver, which may not be practical in real-world applications.

**Table 1:** A comparison between the spiral electrode and four-electrode method^40^.

Item	Four-electrode method^40^		The spiral electrode			
Sample	Ex vivo intact liver (1–2 h of tissues resection)		Tissues (stored at − 80 ℃)		Serum (stored at − 80 ℃)	
Frequency	100 kHz		100 kHz		100 kHz	
Cell type (liver)	Normal	HCC	Normal	HCC	Normal	HCC
Impedance magnitude (Ω)	7 ± 0.5	4 ± 0.5	33.5 ± 0.22	18.5 ± 0.5	29.3 ± 0.34	20 ± 1.2
Conductivity (S/m)	0.091 ± 0.02	0.222 ± 0.07	0.01534 ± 7E−4	0.037 ± 35E−5	0.04 ± 1.47E−3	0.1 ± 7.31E−3
Permittivity	1.1E4 ± 2E3	8.6E3 ± 2E3	2.95E3 ± 35	3.4E3 ± 47	1.28 E3 ± 62	2.8E3 ± 332

## Discussion

The electrochemical impedance spectroscopy (EIS) characterization of serum samples from healthy individuals compared to those from non-healthy individuals highlights the distinction in biological content between these two groups. This difference stems from the varied expression of nucleic acids, protein biomarkers, and extracellular vesicles (such as exosomes, indicative of HCC signature), creating a unique and identifiable signature for HCC compared to the control group, particularly at specified frequencies on our sensor.

Moreover, differentiation between HCC grades (A and D) and cirrhotic/non-cirrhotic samples has been achieved due to their distinct spectra recorded on the sensor. In the subsequent sections, we will provide a detailed description of the key biological differences that serve to distinguish HCC samples from healthy ones.

Hepatocellular carcinoma is a complex heterogeneous disease, with numerous genomic alterations, that negatively affect the physiology of the cell such as uncontrolled cell growth, activation of the oncogenes, inactivation of tumor suppressor genes, and apoptosis escape. In general, the accumulation of genetic aberration with gene mutations that control and govern apoptosis and proliferation is the main cause of developing liver cirrhosis that develops later into cancer. These changes affect the cancer cells’ function and morphology. Liver cancer cells are irregular in shape and lack consistency or fixed pattern concerning the cell size, which appears larger or smaller than normal cells, with multi nucleation, large, nucleus/cytoplasm ratio, and remain immature and unbounded differentiation/proliferation. Also, they depend on anaerobic respiration, which leads to Oxygen deficiency and a hypoxic microenvironment. Consequently, Hypoxia-induced factors will activate hypoxia response genes, promoting the aggressiveness of HCC. Moreover, the extracellular spaces increase and the epithelial structure breaks down, with the cancer tumor invasion and metastasis involving the destruction of the extracellular matrix (ECM) by enzymes such as serine protease, threonine protease, and matrix metalloproteins.

In addition, chronic liver inflammation leads to oxidative stress and lipid peroxidation which is induced by the Reactive Oxygen species (ROS) generated in the mitochondria and negatively influence the regulation of the cell cycle and hence, cell growth, which makes the ROS a main player to the genetic and epigenetic mutations, and lipids peroxidation. Delving into cellular structure, the cytoplasm is regarded as the cell's resistor due to its content of conducting ionic solutions, proteins, nucleic acids, and dissolved organic molecules. Conversely, the thin phospholipid bilayer and the varied protein composition of the cell membrane represent the cell’s capacitance. This composition acts as a dielectric material facilitating a capacitive path with low conductance^34,35^.

Regarding the effect of the ROS (that is accumulated in the cytoplasm in cancer) on the cell’s electrical properties, the cytoplasm’s conductance increases, and it is more soluble in the fluid cell membrane bilayer leading to oxidative damage to the membrane phospholipids and peroxidation of the polyunsaturated fatty acids, in addition to, the increase of oxide composition; during lipid peroxidation^36,37^ that synergistically increase the capacitance of the cell membrane^41^. The evolution of transcriptome science in the previous few years has led to more understanding of the molecular cancer landscape, especially with the improvement and expansion of the Next Generation Sequencing (NGS) technology. Huge networks/databases (The Cancer Genome Atlas (TCGA) and the International Cancer Genome Consortium (ICGC) have been developed and progressed continuously for profiling more than 30 cancers landscape, and cancer transcriptome profile of the coding and non-coding Ribonucleic acid (RNA), with their differential expression in different cancer types in blood and tissues leading to a precise signature for classification cancer types^35,38^. All in all, the genetic variation, cell morphology, immuration and undefinition, nucleus irregular shape, large nucleus/cytoplasm ratio, destruction of the ECM, Hypoxia, ROS, oxide, and lipids peroxidation of the HCC cells, affect the cell's electrical properties and hence, the Impedance, and phase angle measurements.

For a full interpretation of the different behavior between the HCC and normal groups in the tissues and serum and the data consistency between them, some basic knowledge should be mentioned regarding the differences between cancer cells/serum in general (HCC in this study) and normal tissues. This is based on the characteristics and differences between HCC and normal conditions, including gene alterations, and metabolic abnormalities mentioned in the literature^21,27–33^.

### Impedance amplitude and phase angle in cancer and normal tissue

To discuss the difference in impedance and dielectric properties between HCC tissues and normal ones, we are examining the dissimilarities in cellular arrangements between normal liver tissues and HCC tissues. Normal liver tissues exhibit consistent, clear boundaries, a compact structure, and adherence to each other and the extracellular matrix through cell surface proteins. The extracellular spaces are small due to the compact arrangement of normal cells, and fibrous proteins and glycosaminoglycans are expressed normally. Additionally, normal liver tissues mature and differentiate into distinct cell types to perform their functions, with hepatocyte turnover taking a considerable amount of time, almost 6 months.

The disparity in impedance amplitude between HCC and normal tissues is observed, with an increase in HCC tissues and a decrease in normal tissues at low frequencies. These behaviors result in opposition by normal tissues to the electrical current flow, causing an increase in their electrical impedance. However, the properties of cancer tissues mentioned above, such as increased cytoplasm conductance and cell membrane capacitance, lead to a decrease in impedance magnitude and phase angle at low and high frequencies, respectively.

### Impedance amplitude and phase angle in cancer and normal serum samples

The serum data from HCC serum and normal serum show a distinct difference in impedance amplitude while pertaining to the phase angle there is no significant difference. This difference in the spectrum of serum samples between HCC and normal subjects, and their consistency with the cells data, indicates that there is a strong correlation between the cancer cells and the blood components. The variations between the serum contents of the HCC and normal serum samples are attributed to specific biological signatures of the HCC serum sample. The biological content of the HCC serum samples includes different biological biomarkers associated with HCC such as Alpha-Fetoprotein (AFP)^42^, Glypican-3 (GPC3)^43^, Phosphatidylinositol Proteoglycan (GPC-3), Heat Shock Protein (HSP)^44^, Golgi protein-73 (GP73)^45^ and Abnormal Prothrombin (APT)^46^. This high amount of protein biomarkers that existed in HCC serum samples in the high account contributed to the high magnitude of the impedance for HCC over normal samples.

Besides the protein biomarkers, HCC serum is reported with the existence of exosome vesicles which are considered one of the most important extracellular vesicles and considered a signature for each cancer. Exosomes are existing in different body fluids including plasma, urine, and saliva^47^. The main function of exosomes is to facilitate the communication and exchange of substances between cells and is considered a promising source of disease biomarkers^48^. Exosomes are increased in number significantly in the cancer serum and other body fluids compared to the normal serum, which is about 2000 trillion exosomes in normal serum compared to about 4000 trillion in cancer^49–51^. The size of exosomes vesicles is about 50 to 200 nm in diameter and has a cup and/or spherical shape, that emerges from the exocytosis of a multi-vesicular body that forms by the inward budding of an endosomal compartment^52–54^. They originated from the parental cancer cells (HCC in this study), and are considered as a fingerprint to these cells, by having the genetic information of their originated cells, including distinguished cargos such as coding and non-coding RNAs, DNA, proteins, lipids, and carbohydrates.

Both exosomes and protein biomarkers have demonstrated their ability to distinguish between different cancer types in the study. To detect exosomes and their cargos, numerous researchers have focused on developing biosensors using diverse approaches such as microfluidics, nanoparticle-based techniques, and electrochemical methods. These methods are chosen based on the unique signature produced by exosomes when interacting with capacitance sensors. Recently, Lee et al.^55^ have developed a capacitance-based biosensor for the detection of exosomes in undiluted serum. Despite the high performance of the developed sensor, in identifying and quantifying the exosomes from different sources, they have used Deoxyribonucleic acid (DNA) aptamer/Molybdenum disulfide (MoS2) on the sensor for capturing exosomes (Aptamer/CD63 on exosomes and Molybdenum disulfide (MoS2) for enhancing the electrical sensitivity. Moreover, an article by Ahmad^56^ for the detection, and quantification of exosomes in different cell lines, based on voltage applying to the exosomes and measuring their capacitance–voltage profile. According to the author, the detection technique is based on applying a voltage to the exosome vesicles, an interface layer is formed due to the interaction between the exosome’s surface and its surrounding media. The study has concluded that the capacitance variabilities in the different cell lines depend on the exosome’s type, cargo, and morphology providing fingerprint signature for different types of exosomes and thus, influence the exosome polarization and their capability to hold charges, which reflects the parent cells that produce the exosomes. Moreover, they have found that the capacitance decreases as the number/count of exosomes decreases, and this change was due to the difference in the exosomes count. Despite, the high accuracy, specificity, and sensitivity of the identification and quantification of the exosomes, the method has been done on exosomes produced from cell lines, not real clinical samples in addition, an extraction step for the exosomes has been done, which increase the cost and time of the detection method.

However, the phase angle observed in serum samples from both normal and HCC groups does not display notable differences. The serum composition, containing protein biomarkers and exosomes ranging from 50 to 200 nm in size, represents a relatively smaller fraction compared to tissues or larger particles like cells, typically ranging from 10 to 2 µm. This discrepancy in size may contribute to the absence of significant phase angle variations between the healthy and HCC groups. The phase angle tends to notably increase with larger particle sizes, as seen in cells, while exosomes, due to their smaller size, may not generate a sufficiently noticeable impact on the phase angle values. Moreover, the discrepancy in impedance amplitude and phase angle is statistically significant between normal and HCC tissue samples, while it is less pronounced between normal serum and HCC serum samples. As described earlier, this difference is primarily due to variations in the structure of normal and HCC tissues, such as differences in cell membrane and extracellular matrix (ECM). In serum samples, the difference is mainly attributed to blood components such as proteins, biomarkers, and exosomes. The relatively smaller size of blood components compared to tissues and cells results in a less pronounced statistical difference between normal and HCC serum samples, whereas a more significant statistical difference is observed between normal and HCC tissue samples.

### Cirrhosis and BCLC stage measurements

As shown in Fig. 6a, cirrhotic liver samples were compared to non-cirrhotic liver samples using the proposed spiral EIS efficiency in cirrhosis detection. The samples with cirrhosis have a greater impedance amplitude than the samples without cirrhosis. This means that when fibrosis progresses, cell structure changes occur (e.g.: scars) and resistance to the current flow takes place hence, affecting the current flow and leading to an increase in the impedance amplitude.

In Fig. 6b, the BCLC stage has also been tested with the designed spiral EIS. Interestingly, samples in stage D have a lower impedance amplitude than the samples in stage A, this indicates that as the BCLC stage develops, cell activity increases, resulting in changes in the cell structure and components as mentioned before, and a small and higher impedance magnitude observed in the lower and higher BCLC Stage respectively.

## Conclusion

This study presents a novel spiral sensor utilizing impedance spectroscopy and a unique PCB design with high surface area electrodes. This sensor demonstrates exceptional promise for detecting unlabeled HCC in clinical samples, distinguishing between cells and serum components rapidly and efficiently due to its ability to measure capacitance and impedance accurately. The distinct electrical fingerprint of HCC samples in serum, driven by unique biomarkers like protein biomarkers, nucleic acids, and extracellular vesicles, showcases its potential for serum-based diagnostics. Variations in biomarker secretion, exosomes, cell membrane profiles, and HCC cell sizes significantly influence impedance variations. Moreover, the spiral sensor effectively characterizes cirrhosis and BCLC stages by leveraging the distinctive impedance properties of characteristic cells. Its proficiency in distinguishing between serum and cell specimens from HCC and normal sources highlights its consistent and accurate data acquisition capabilities.

Additionally, the chip’s ability to differentiate between different HCC stages suggests its potential as a rapid prescreening tool. This could streamline diagnostics, offering a cost-effective alternative to existing, time-consuming techniques. Ultimately, this innovative spiral sensor holds promise for revolutionizing HCC diagnosis and impacting the broader landscape of cancer detection and characterization.

The spiral sensors based on EIS can serve as point-of-care tools for distinguishing between normal liver tissues and serum from HCC tissues, as well as differentiating normal liver tissues from cirrhotic ones. This technology has the potential to transform cancer characterization by providing insights into dielectric property variations across different cancer types and clinical disorders. Ongoing research aims to establish specific frequency-based signatures for various cancer types, improving diagnostic precision.

### Supplementary Information

Supplementary Information.

## Data Availability

The datasets used and analyzed in this study are available from the corresponding author upon reasonable request. All the data from lab tests for the patient can be found in the Supplementary Information.

## Abbreviations

HCC: Hepatocellular carcinoma
PCB: Printed circuit board
EIS: Electrical impedance spectroscopy
AC: Alternating current
BCLC: Barcelona clinic liver cancer
NLT: Normal liver tissues
ECM: Extracellular matrix
ROS: Reactive oxygen species
TCGA: The cancer genome atlas
ICGC: International cancer genome consortium
RNA: Ribonucleic acid
DNA: Deoxyribonucleic acid
MoS2: Molybdenum disulfide
