# Supplementary material for: A new label free spiral sensor using impedance spectroscopy to characterize hepatocellular carcinoma in tissue and serum samples
Source: Sci Rep. 2024 Jun 7;14:13155. doi: 10.1038/s41598-024-63141-5 (PMC11161506; doi:10.1038/s41598-024-63141-5)
Supplement: Supplementary file 1 — Supplementary Information. [file 41598_2024_63141_MOESM1_ESM.docx]

**Development of A New Spiral Electro-Impedance Spectroscopy as a label-free sensor for characterizing Hepatocellular Carcinoma in both tissue and serum samples.**

Reda Abdelbaset ^1,2,^ , Sherif M. Shawky ^3,4^, Mohammed A. A. Abdullah ^* 2^, Omar E. Morsy ^2^, Yehya H. Ghallab ^1,2^*,* Marwa M. Sayed^5^, and Yehea Ismail^2^.

^1^ Biomedical Engineering Department, Helwan University, Cairo, Egypt.
^2^ Centre of Nanoelectronics and Devices (CND), Zewail City of Science and Technology and The American University in Cairo (AUC), Egypt.

^3^ Faculty of Pharmacy, Biochemistry Department, Misr University for Science and Technology, Giza, Egypt.

^4^ Center of Genomics, Helmy Institute, Zewail City of Science and Technology, Giza, Egypt.

^5^ Medical Biochemistry and Molecular Biology Department, Faculty of Medicine, Ain Shams University, Cairo, Egypt.

Corresponding author: Mohammed A. A. Abdullah (mohammed.abdullah@alumni.albany.edu)

Supplementary information

**Table 1: Demographic and lab data of participants**

| **S** | **Group** | **sex** | **age** | **AST** | **ALT** | **T.bilirubin** | **D.bilirubin** | **Albumin** | **INR** | **HCV.ABS** | **Cirrhosis** | **BCLCstage** | **AFP** | **Tumour**  **Size** | **HBV.AG** |
| --- | --- | --- | --- | --- | --- | --- | --- | --- | --- | --- | --- | --- | --- | --- | --- |
| **1** | **HCC** | **male** | **53** | **100** | **63** | **1.5** | **0.6** | **2.6** | **1.4** | **positive** | **Yes** | **A** | **350** | **2.6X2.4** | **negative** |
| **2** | **HCC** | **male** | **60** | **58** | **43** | **1.3** | **0.6** | **3.2** | **1.2** | **positive** | **No** | **A** | **10.8** | **1.5x2** | **negative** |
| **3** | **HCC** | **female** | **60** | **103.8** | **73** | **1.5** | **0.8** | **2.4** | **1.5** | **positive** | **Yes** | **D** | **355** | **3x2** | **negative** |
| **4** | **HCC** | **male** | **65** | **39** | **31** | **1.76** | **0.9** | **2.7** | **1.6** | **positive** | **Yes** | **A** | **73.8** | **4x3** | **negative** |
| **5** | **HCC** | **female** | **60** | **60** | **32** | **1** | **0.35** | **2.9** | **1.3** | **positive** | **Yes** | **A** | **1200** | **4x3** | **negative** |
| **6** | **HCC** | **female** | **50** | **102** | **67** | **1.8** | **0.5** | **3.1** | **1.45** | **positive** | **Yes** | **A** | **136** | **1.5x2** | **negative** |
| **7** | **HCC** | **male** | **52** | **152** | **95** | **2.4** | **0.9** | **3.1** | **1.5** | **positive** | **Yes** | **A** | **20.5** | **2x2** | **negative** |
| **8** | **HCC** | **male** | **69** | **48** | **37** | **2** | **0.8** | **3** | **1** | **positive** | **Yes** | **A** | **4** | **4X4** | **negative** |
| **9** | **HCC** | **male** | **60** | **33** | **42** | **1.6** | **0.6** | **3.2** | **1.2** | **positive** | **No** | **A** | **1,985** | **3x3** | **negative** |
| **10** | **HCC** | **male** | **64** | **52** | **19** | **3.9** | **2.2** | **3.2** | **1.3** | **negative** | **No** | **A** | **23.4** | **4x5** | **positive** |
| **11** | **HCC** | **male** | **67** | **40** | **26** | **2.5** | **0.9** | **2.7** | **1.4** | **positive** | **Yes** | **A** | **73** | **2.8X3** | **negative** |
| **12** | **HCC** | **female** | **37** | **46** | **33** | **1** | **0.5** | **2.9** | **1.5** | **positive** | **Yes** | **A** | **2.3** | **2x2.3** | **negative** |
| **13** | **HCC** | **female** | **53** | **43** | **14** | **2.3** | **1.9** | **3** | **1.4** | **positive** | **Yes** | **A** | **2.9** | **2x2** | **negative** |
| **14** | **HCC** | **male** | **58** | **34** | **85** | **1.8** | **1.2** | **2.9** | **1.32** | **positive** | **Yes** | **A** | **3,224** | **4X3** | **negative** |
| **15** | **HCC** | **female** | **64** | **104** | **56** | **2.6** | **1.1** | **2.6** | **1.36** | **positive** | **Yes** | **A** | **468** | **4X5** | **negative** |
| **16** | **HCC** | **male** | **61** | **102** | **37** | **1** | **1** | **3** | **1** | **positive** | **Yes** | **A** | **10** | **4X5** | **negative** |
| **17** | **HCC** | **male** | **52** | **100** | **55** | **2.8** | **1.5** | **3** | **1.3** | **positive** | **Yes** | **A** | **6.7** | **2.5X2** | **negative** |
| **18** | **HCC** | **male** | **57** | **23** | **17** | **1.6** | **1.2** | **3** | **1.29** | **positive** | **Yes** | **A** | **3.3** | **3.4X3.4** | **negative** |
| **19** | **HCC** | **female** | **67** | **127** | **78** | **0.6** | **0.3** | **2.9** | **1.28** | **positive** | **Yes** | **A** | **22** | **4X3** | **negative** |
| **20** | **HCC** | **female** | **65** | **38** | **28** | **0.6** | **0.1** | **4.1** | **1.05** | **negative** | **No** | **A** | **6** | **--** | **negative** |
| **21** | **HCC** | **female** | **50** | **62** | **74** | **1.5** | **0.5** | **3.2** | **1.8** | **positive** | **Yes** | **A** | **33** | **3X3.5** | **negative** |
| **22** | **HCC** | **male** | **52** | **48** | **53** | **1.1** | **0.5** | **3.1** | **1.33** | **negative** | **Yes** | **A** | **11** | **2.5X3** | **negative** |
| **23** | **HCC** | **male** | **57** | **38** | **35** | **1.2** | **0.7** | **3** | **1.5** | **negative** | **Yes** | **A** | **8.2** | **2X2** | **negative** |
| **24** | **HCC** | **male** | **47** | **108** | **90** | **4.9** | **2.4** | **2.2** | **1.6** | **positive** | **Yes** | **D** | **21** | **1.4X4** | **negative** |
| **25** | **Control** | **female** | **56** | **68** | **77** | **1.4** | **0.7** | **3.4** | **1.09** | **positive** | **Yes** | **--** | **7** | **--** | **negative** |
| **26** | **Control** | **male** | **55** | **42** | **43** | **2** | **1** | **3.3** | **1.2** | **positive** | **No** | **--** | **2** | **--** | **negative** |
| **27** | **Control** | **female** | **57** | **56** | **29** | **2.9** | **1** | **2.9** | **1.32** | **positive** | **No** | **--** | **2** | **--** | **negative** |
| **28** | **Control** | **male** | **54** | **42** | **22** | **1.6** | **0.6** | **2.7** | **1.25** | **positive** | **Yes** | **--** | **8** | **--** | **negative** |
| **29** | **Control** | **male** | **62** | **82** | **37** | **3.1** | **1.8** | **2.9** | **1.4** | **positive** | **Yes** | **--** | **7** | **--** | **negative** |
| **30** | **Control** | **female** | **46** | **91** | **19** | **12.2** | **10.2** | **3.3** | **1.2** | **negative** | **No** | **--** | **3** | **--** | **negative** |
| **31** | **Control** | **female** | **58** | **68** | **47** | **3.6** | **2.1** | **3.1** | **1.16** | **positive** | **Yes** | **--** | **4** | **--** | **negative** |
| **32** | **Control** | **male** | **50** | **45** | **27** | **0.9** | **0.4** | **2.7** | **1.28** | **positive** | **Yes** | **--** | **5** | **--** | **negative** |
| **33** | **Control** | **female** | **54** | **17** | **11** | **1.1** | **0.1** | **3.6** | **1.1** | **negative** | **No** | **--** | **7** | **--** | **negative** |
| **34** | **Control** | **female** | **58** | **33** | **31** | **0.52** | **0.05** | **4.3** | **1.1** | **negative** | **No** | **--** | **2** | **--** | **negative** |
| **35** | **Control** | **female** | **50** | **19** | **22** | **1** | **0.2** | **4** | **1.1** | **negative** | **No** | **--** | **2** | **--** | **negative** |
| **36** | **Control** | **female** | **55** | **18** | **20** | **1.1** | **0.1** | **3.9** | **1.1** | **negative** | **No** | **--** | **12** | **--** | **negative** |
| **37** | **Control** | **male** | **45** | **25** | **28** | **1.1** | **0.3** | **4.1** | **0.9** | **negative** | **No** | **--** | **23** | **--** | **negative** |


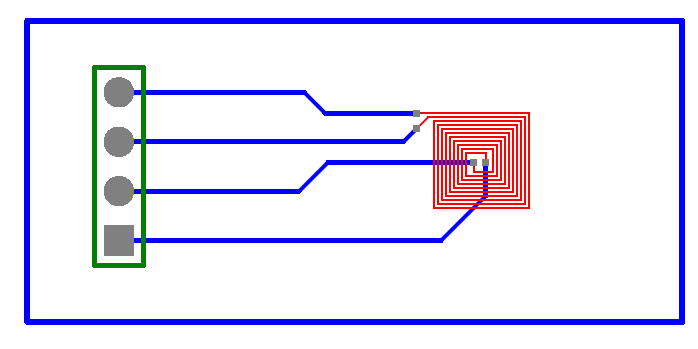


Figure 1. Snapshot of CAD file for the sensor design
